# A Mosaic from Louis Pasteurís Crypt

**DOI:** 10.3201/eid0503.AC0503

**Published:** 1999


					Publisher: CDC; Journal: Emerging Infectious Diseases
Article Type: About the Cover; Volume: 5; Issue: 3; Year: 1999; Article ID: AC-0503
DOI: 10.3201/eid0503.AC0503; TOC Head: About the Cover
Page 1 of 1
Suggested citation: A Mosaic from Louis Pasteuris Crypt [about the cover]. Emerg Infect
Dis [serial on the Internet]. 1999 Jun [date cited].http://dx.doi.org/10.3201/eid0503.AC0503
Polyxeni Potter, EID Journal, Centers for Disease Control and Prevention, 1600 Clifton Rd, Mailstop D61,
Atlanta, GA 30333, USA; email: PMP1@cdc.gov
A Mosaic from Louis Pasteuris Crypt
A mosaic from Louis Pasteuris crypt (1896, Institut Pasteur) representing the well-known
episode of the shepherd Jean-Baptiste Jupille struggling against a rabid dog. The shepherd was
the second person ever to be vaccinated against rabies. The crypt, modeled on a Byzantine
mausoleum, was decorated in a symbolist style by noted French artists of the end of the 19th
century. Each mosaic depicts one of Pasteuris main accomplishments (rabies, silkworms, wine,
veterinary vaccines). Louis Pasteur rests in the crypt located in the Institut Pasteur in Paris.
Figure. A mosaic from Louis Pasteuris crypt (1896, Institut Pasteur) Printed with permission of the Institut
Pasteur.

				

A mosaic from Louis Pasteurís crypt (1896, Institut Pasteur) representing the well-known episode of the shepherd Jean-Baptiste Jupille struggling against a rabid dog. The shepherd was the second person ever to be vaccinated against rabies. The crypt, modeled on a Byzantine mausoleum, was decorated in a symbolist style by noted French artists of the end of the 19th century. Each mosaic depicts one of Pasteurís main accomplishments (rabies, silkworms, wine, veterinary vaccines). Louis Pasteur rests in the crypt located in the Institut Pasteur in Paris. 

**Figure:**
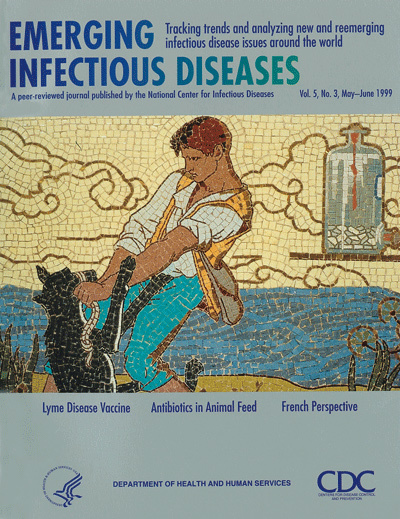
A mosaic from Louis Pasteurís crypt (1896, Institut Pasteur) Printed with permission of the Institut Pasteur.

